# Sexual dimorphism in atrophic effects of topical glucocorticoids is driven by differential regulation of atrophogene REDD1 in male and female skin

**DOI:** 10.18632/oncotarget.27445

**Published:** 2020-01-28

**Authors:** Gleb Baida, Shivani Agarwal, Ben Readhead, Joel T. Dudley, Irina Budunova

**Affiliations:** ^1^Feinberg School of Medicine, Department of Dermatology, Northwestern University, Chicago, IL, USA; ^2^Icahn School of Medicine at Mount Sinai, New York, NY, USA; ^3^Current address: ASU-Banner Neurodegenerative Disease Research Center, Arizona State University, Tempe, AZ, USA

**Keywords:** glucocorticoid, skin atrophy, REDD1, mTOR, sexual dimorphism

## Abstract

Topical glucocorticoids, well-known anti-inflammatory drugs, induce multiple adverse effects, including skin atrophy. The sex-specific effects of systemic glucocorticoids are known, but sexual dimorphism of therapeutic and side effects of topical steroids has not been studied. We report here that female and male mice were equally sensitive to the anti-inflammatory effect of glucocorticoid fluocinolone acetonide (FA) in ear edema test. At the same time, females were more sensitive to FA-induced skin atrophy. We recently reported that REDD1 (regulated in development and DNA damage 1) plays central role in steroid atrophy. We found that REDD1 was more efficiently activated by FA in females, and that REDD1 knockout significantly protected female but not male mice from skin atrophy. Studies using human keratinocytes revealed that both estradiol and FA induced REDD1 mRNA/protein expression, and cooperated when they were combined at low doses. Chromatin immunoprecipitation analysis confirmed that REDD1 is an estrogen receptor (ER) target gene with multiple estrogen response elements in its promoter. Moreover, experiments with GR and ER inhibitors suggested that REDD1 induction by these hormones was interdependent on functional activity of both receptors. Overall, our results are important for the development of safer GR-targeted therapies suited for female and male dermatological patients.

## INTRODUCTION

Glucocorticoids (GCs) are important regulators of skin cell proliferation, differentiation, and immune response. They are synthesized in adrenal glands but also in peripheral tissues including skin [1–3], and their biosynthesis is tightly regulated [4, 5]. Synthetic GCs are among the most effective and frequently used anti-inflammatory drugs for different inflammatory and autoimmune skin diseases, including atopic dermatitis and psoriasis. Unfortunately, chronic treatment with GCs results in multiple deleterious side effects, including skin atrophy.

Skin atrophy involves all skin compartments and is characterized by a dramatic loss in skin thickness, increased fragility, tearing, bruising, and results in compromised skin barrier function, followed by the increased risk for developing secondary wounds and infections at the affected site [6–10]. Although GCs-induced skin atrophy is well described at the phenotypic level, the underlying molecular mechanisms just started to emerge. Recently we and others identified mTOR/Akt inhibitor REDD1 (regulated in development and DNA damage 1) as the key mediator of GCs-induced atrophy in skin and muscle. Indeed, in REDD1 KO animals muscle as well as all skin compartments: epidermis, dermis, and dermal adipose were protected against GCs-induced atrophy [11, 12].

It is well known that sex steroid hormones profoundly affect various aspects of normal skin morphology, physiology, as well as risk for certain cutaneous diseases. For example, sex steroids affect skin thickness, skin surface pH, wound healing [13, 14]. Women exhibit a higher prevalence of inflammatory and autoimmune skin and other diseases such as rosacea, lupus, and scleroderma [15–17]. Interestingly, in murine skin, estrogens play an important role in the control of epidermis and dermal adipose, while dermis is controlled by androgens [13]. In humans, it is well documented that thinning of the skin and loss of dermal collagen occur with the loss of circulating estrogens in postmenopausal women [18, 19].

The sex-specific effects of systemically administered GCs on immune and central nervous systems, and liver are known, and this dimorphism is linked to the differential gene expression regulation by GCs in males and females [16, 17, 20]. These previous findings indicated that males are overall more sensitive to the anti-inflammatory effect of systemic GCs than females. However, the sex dependence of therapeutic and side effects of topical steroids has not been documented or explored, even though it is pertinent to the treatment regimens optimization in patients. Indeed, currently, topical (as well as systemic) glucocorticoids are prescribed at similar doses and regiments to men and women. In fact, until recent years, only few dermatological research papers compared the effects of different compounds including hormones in male versus female cells or animals, and the vast majority of authors either do not disclose sex or use only one sex in their work [15].

The goal of studies presented here was to compare the response of male and female mice to anti-inflammatory and atrophogenic effects of topical GCs, and to further corroborate the role of atrophogene REDD1 in skin with a focus on sexually dimorphic response to these steroid hormones.

We found that male and female mice were equally sensitive to the anti-inflammatory effect of topical glucocorticoid fluocinolone acetonide (FA) in the ear edema test. Unexpectedly females appeared more sensitive to FA-induced skin atrophy, especially when lower doses of glucocorticoid fluocinolone acetonide (FA) were used. This was in part due to more efficient REDD1 induction in female skin. Moreover, knockout (KO) of REDD1 protected females but not males against atrophic side effects of topical GCs. REDD1 is a known glucocorticoid receptor (GR) target gene [21–23, 12]. Here we report that estrogen receptor (ER) also controls REDD1 expression, and that GCs and estrogens cooperated in the induction of REDD1 in keratinocytes.

## RESULTS

### Male and female mice are equally sensitive to the anti-inflammatory effects of glucocorticoids

**Figure 1:**
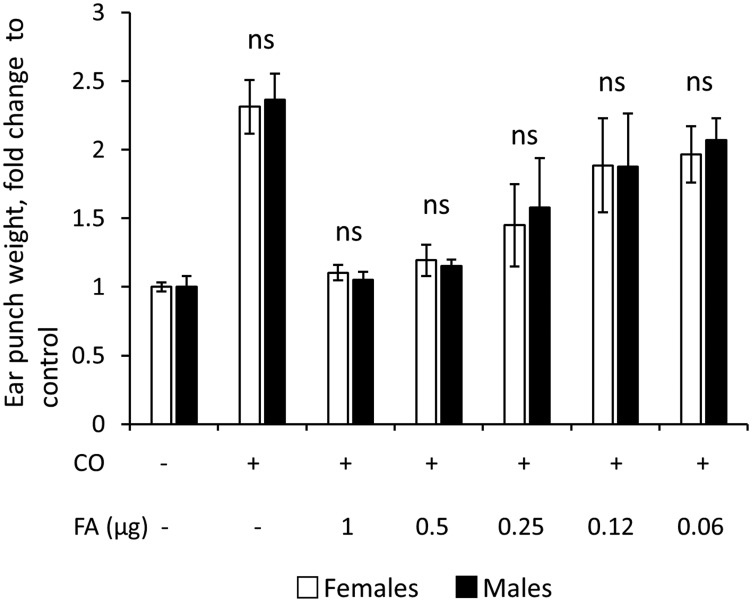
Similar sensitivity of males and females to inflammation and anti-inflammatory effects of glucocorticoid FA in ear edema test. Ear edema was induced by croton oil (CO) in 7-week-old female and male wild-type F1 B6 × 129 mice as in Materials and Methods. FA was applied 1 h before CO, and five-millimeter ear punches were weighed 9 h after CO application to assess swelling. Results are presented as fold changes to corresponding (female or male) control ear weight. The means ± SD were calculated for six individual ear punches/condition in one representative experiment. The unpaired two-tailed *t*-test was used for statistical analysis for differences between males and females in the same treatment groups. In all cases the differences were non-significant (ns, *P* > 0.3).

In order to quantitatively assess the anti-inflammatory effects of glucocorticoids, we employed a widely used ear edema test [24]. The inflammation was induced by topical application of skin irritant croton oil (5% in 20 µl acetone) to the ears of F1 B6 × 129 wild-type mice, and ear edema (measured 9 h later by ear punch weight) was used as the readout for general inflammatory response. The glucocorticoid FA was applied 1 h before the croton oil. As shown in Figure 1, male and female mice showed equal edema after croton oil and were equally sensitive to FA-induced inhibition of ear inflammation over a broad range of doses with complete inhibition of swelling at 1 µg FA for both sexes. Thus, we found no sexual dimorphism in the induction of inflammation or in the anti-inflammatory effects of glucocorticoid FA in the skin.

### Females are more sensitive to skin atrophy induced by topical glucocorticoids due to the more effective induction of atrophogene REDD1

**Figure 2:**
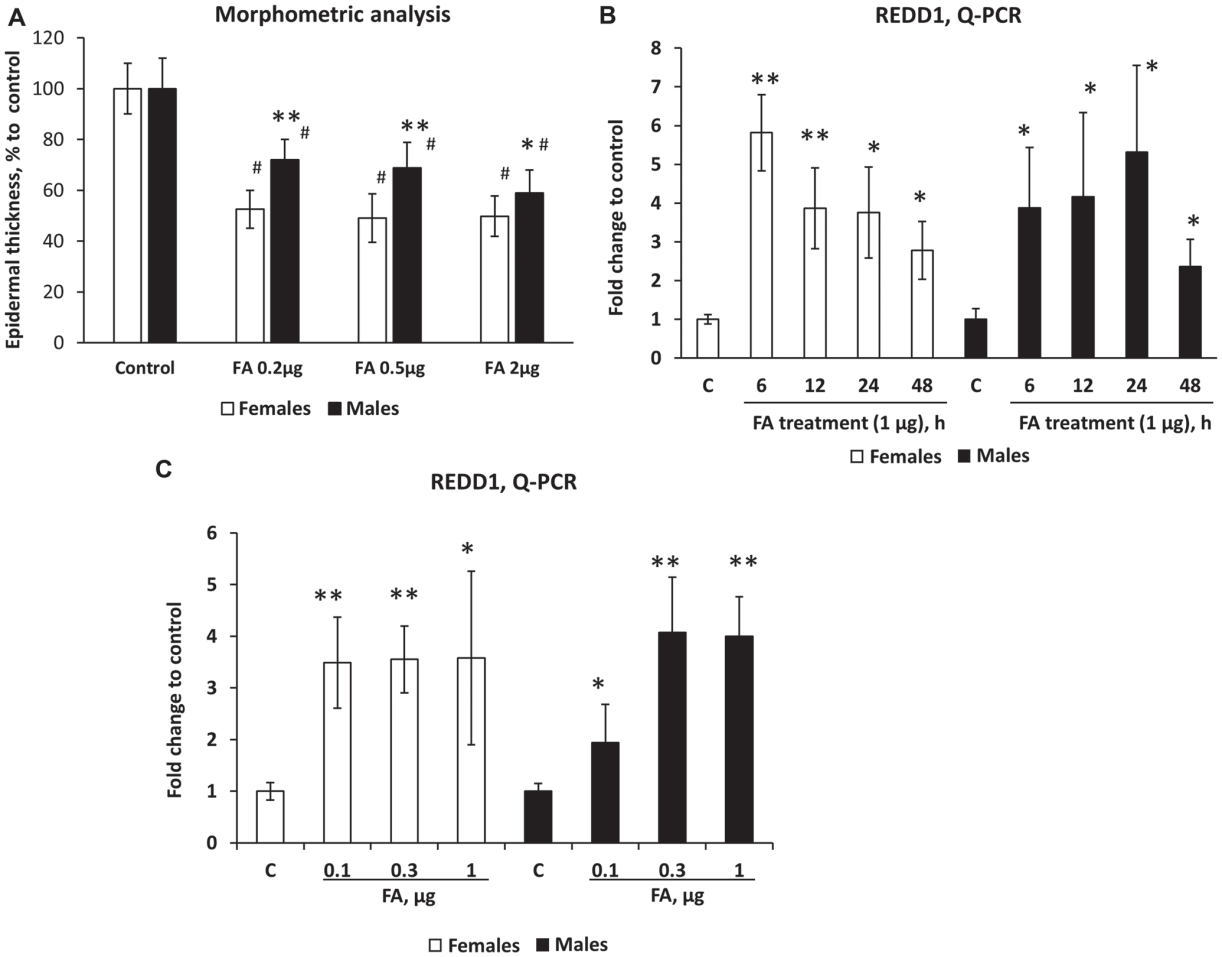
Females are more sensitive than males to REDD1 induction and glucocorticoid FA-induced skin atrophy. Seven-week-old female and male wild-type F1 B6 × 129 mice were treated topically with vehicle (Control, 200 µl acetone) or FA (at indicated doses) every 72 h for 2 weeks (**A**) or once for 6–48 h (**B**) or once for 24 h (**C**). (A) Epidermal atrophy induced by chronic FA applications was assessed by morphometric analysis of epidermal thickness in H&E skin sections as in Materials and Methods. Changes in epidermal width are presented as % to corresponding control epidermis. The means ± SD were calculated for three individual skin samples per condition in one representative experiment (totally 30 measurements/condition). The unpaired two-tailed *t*-test was used for statistical analysis: ^*^ *P* < 0.01, ^**^ *P* < 0.0001, for differences between atrophy in males compared to females in the same treatment group; ^#^ *P* < 0.0001, for changes compared to corresponding controls. (B and C) REDD1 mRNA expression in mouse epidermis was measured by Q-PCR as in Materials and Methods. Rpl27 was used as a cDNA normalization control. Q-PCR results are the means ± SD calculated for three individual RNA samples/condition. Statistical analysis for differences between treatment and corresponding control was done by the unpaired two-tailed *t*-test. ^*^ *P* < 0.05; ^**^ *P* < 0.01.

An undesirable side effect of chronic topical glucocorticoid treatment is skin atrophy. To assess whether there is a sexually dimorphic response to steroid-induced atrophy, we used the previously developed skin atrophy model [12, 25]. Skin hypoplasia was induced by the range of FA doses (0.2–2 µg/animal) applied dorsally twice a week for 2 weeks. As epidermis is considered the most sensitive skin compartment in terms of atrophic response, and the measurements of epidermal thickness were included in the test system for steroid skin atrophy [8, 26], we used morphometric analysis of epidermal thickness as the readout for development of skin atrophy. Our experiments revealed that epidermis in female mice was more sensitive to FA atrophogenic effect at low doses (0.2–0.5 µg, Figure 2A). However, at higher (2 µg) dose FA was equally effective in the induction of skin atrophy in mice of both sexes (Figure 2A). It is known that dermal adipose is also very sensitive to topical glucocorticoids, and could undergo severe atrophy until almost complete reduction during chronic topical treatment [9, 12]. Interestingly, the analysis of FA effect on dermal adipose at low doses, including 0.04 µg, the dose that practically did not affect epidermis, confirmed higher sensitivity of females to atrophic effects of glucocorticoids (Supplementary Figure 1). The quantitative analysis of dermal adipose at higher doses was not possible due to the loss of most part of this fat depot.

We have recently identified several atrophogenes, genes that are required for the induction of skin atrophy by glucocorticoids, including mTOR/Akt inhibitor REDD1 [12, 27]. We reported earlier that REDD1 is strongly induced in mouse and human skin by glucocorticoids and its knockout renders mice resistant to glucocorticoid-induced skin atrophy [12]. We show here that REDD1 was induced by FA earlier and more efficiently at low doses in the female epidermis (Figure 2B, 2C), which correlated well with higher sensitivity of females to steroid skin atrophy.

**Figure 3:**
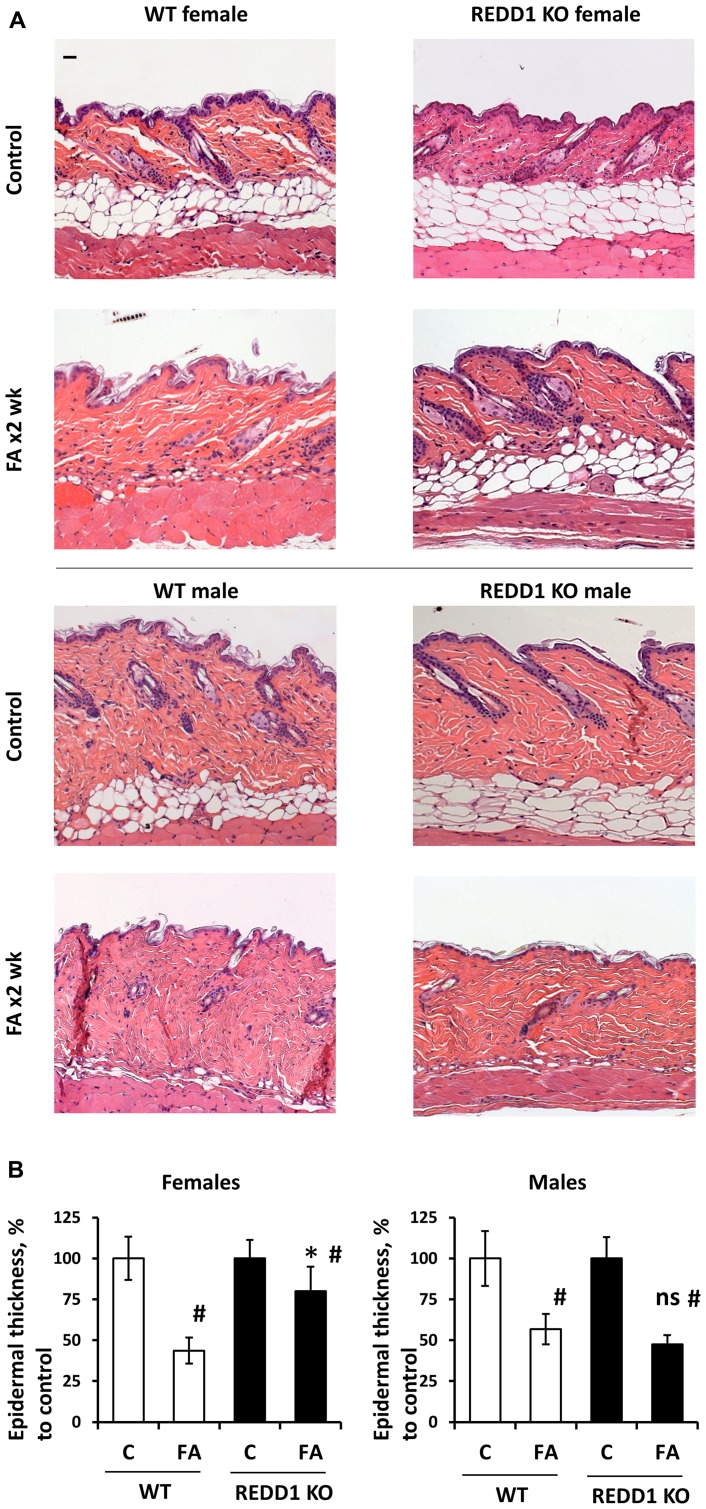
REDD1 KO female but not male mice are protected against glucocorticoid FA-induced skin atrophy. Seven-week-old F1 B6 × 129 WT and REDD1 KO male and female mice were treated with vehicle (Control, 200 µl acetone) or FA (2 µg/animal) every 72 h for 2 weeks. (**A**) Formalin-fixed, paraffin-embedded skin sections stained with hematoxylin and eosin. Scale bars are 20 µm. (**B**) Epidermal thickness in female and male WT and REDD1 KO mice was evaluated as in Materials and Methods. Epidermal thickness is presented as % to corresponding (sex/genotype) control epidermis. The means ± SD were calculated for three individual skin samples in one representative experiment (totally 30 measurements/condition). The unpaired two-tailed *t*-test was used for statistical analysis: ^*^ *P* < 0.001, for differences between atrophy in WT and REDD1 KO females; ns, (*P* > 0.3) for differences between atrophy in WT and REDD1 KO males; ^#^ *P* < 0.001, for changes compared to corresponding controls. Note: Only in REDD1 KO female epidermis and dermal adipose were significantly protected against FA-induced hypoplasia.

Our previous observation that knockout of REDD1 provided a protective effect from FA-induced skin atrophy was done in female mice [12]. We repeated these experiments in female and male REDD1 KO and isogenic wild-type animals. As REDD1 KO animals have mild epidermal hyperplasia and more extensive dermal adipose [12], we evaluated skin hypoplasia via comparison of FA-treated animals with corresponding (sex/genotype) vehicle control groups (Figure 3A). Chronic FA treatment (2 µg twice a week for 2 weeks) resulted in ~40% thinning of the epidermis in wild-type males and ~50% in KO males (Figure 3B). At the same time, the epidermal thickness was reduced by more than 50% in wild-type females, but only by 20% in REDD1 KO females. The similar sex-depended effect was observed in dermal adipose, which was protected from FA-induced atrophy only in female REDD1 KO animals (Supplementary Figure 2). Thus, knockout of REDD1, protective in female skin, did not significantly spare skin from glucocorticoid-induced atrophy in males.

### REDD1 is the ER target gene, and its expression is co-regulated by glucocorticoids and estrogens

The results of our *in vivo* experiments suggested that the potential cross-talk between estrogen- and glucocorticoid-induced signaling may play an important role in the regulation of REDD1 expression in skin. To examine the potential effects of steroid hormones on REDD1 expression, we used human immortalized keratinocyte cell line HaCaT. In these cells, GR is well expressed and highly functional [12, 23]. They also express estrogen receptors, ERα and ERβ, which mediate estrogen signaling, as well as androgen receptor, AR [28, 29].

**Figure 4:**
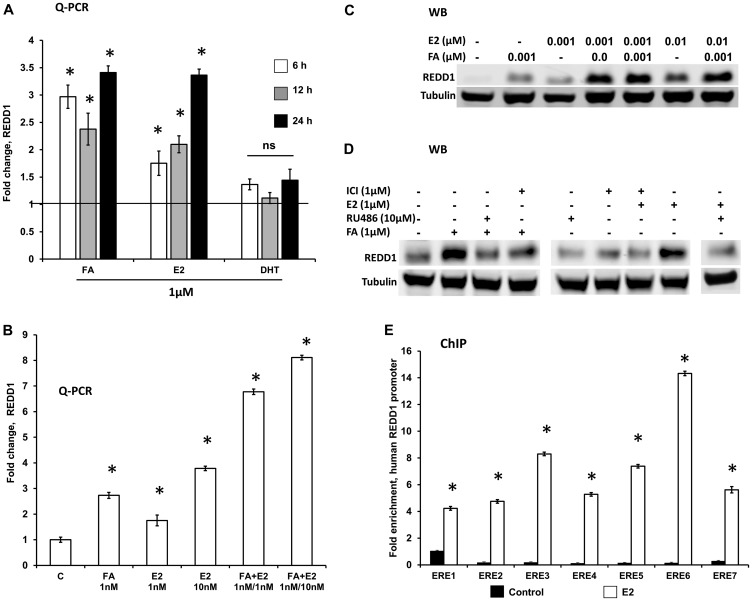
REDD1 expression in keratinocytes is co-regulated by glucocorticoids and estrogens. Immortalized human keratinocytes HaCaT were switched to hormone-free medium for 24 h as in Materials and Methods and treated with steroid hormones and inhibitors as detailed below. (**A**) Q-PCR analysis of REDD1 induction by glucocorticoid FA, estradiol (E_2_), and androgen DHT (1 µM for 6–24 h). (**B** and **C**) E_2_ and FA cooperatively induced REDD1 expression at low concentrations (1 nM–10 nM for 12 h), as detected by Q-PCR (B) and Western blotting (C). ^*^ *P* < 0.01, for changes compared to control, two-tailed unpaired *t*-test (A and B). (**D**) Western blot analysis of REDD1 expression in HaCaT cells pre-incubated for 6 h with GR inhibitor RU486 or ER inhibitor ICI182,780 (Fulvestrant), and then treated with either FA or E_2_ for 24 h. Control cells were treated with solvent only (0.1% Etoh). Tubulin was used as a loading control. (**E**) ChIP analysis of ER loading on REDD1 promoter. HaCaT cells grown in steroid-free medium as in Materials and Methods, were treated with a vehicle (control, 0.01 EtOH) or estradiol (E_2_, 1 µ) for 24 h and processed for ChIP (3 independent experiments) as in Materials and Methods. ^*^ *P* < 0.001, for changes compared to control, two-tailed unpaired *t*-test.

REDD1 is a well-known GR target gene with multiple GR-binding sites in its promoter [21–23, 30]. As expected, glucocorticoid FA robustly induced REDD1 expression in 6–24 h (Figure 4A). Interestingly, estrogen (E_2_) was also able to significantly induce REDD1 expression, while AR ligand dihydrotestosterone (DHT) had little effect on REDD1 levels (Figure 4A). In order to determine if glucocorticoids and estrogens may act cooperatively, HaCaT cells were treated with fairly low (10^–9^ M) doses of FA and E_2_ that were minimally effective for REDD1 induction when used alone. However, the combination of FA and E_2_ resulted in a much greater induction of REDD1 at both mRNA (Figure 4B) and protein (Figure 4C) levels compared to the single hormone treatment.

To further assess the role of GR/ER crosstalk in cooperative REDD1 regulation by steroids, we blocked GR and ER functions using their antagonists RU0486 (for GR) and Fulvestrant (for ER). Reflecting the known role of GR in REDD1 gene expression regulation, RU0486 alone significantly blocked basal REDD1 expression (Figure 4D), while Fulvestrant had only borderline effect on REDD1 basal level. Interestingly, the pre-treatment of cells with RU0486 blocked not only GR-dependent REDD1 induction but E_2_-induced REDD1 induction as well (Figure 4D). Similarly, ER antagonist, Fulvestrant, blunted both E_2_- and FA-induced REDD1 induction (Figure 4D), suggesting that REDD1 induction in response to these steroid hormones is interdependent on functional activity of both these receptors.

To further understand the mechanism of REDD1 induction by E_2,_ we assessed whether ER loads on estrogen response elements (EREs) in the promoter region of the human REDD1 gene. Putative ER binding sites in the human REDD1 promoter were predicted based on HOCOMOCO models of TF binding motifs [31] as described in Materials and Methods. We used ChIP analysis of seven putative ER binding sites and found that ER indeed occupied all these predicted sites in cells treated with E_2_, thereby explaining the possible mechanism of REDD1 induction by estrogens (Figure 4E).

## DISCUSSION

The major goal of this work was to evaluate whether and how the responses to anti-inflammatory and adverse effects of topical GCs depend on sex in animal models of dermatitis and skin atrophy. We found that the anti-inflammatory effect of glucocorticoid FA was similar in male and female mice in the ear edema test. This result is different from the previously reported larger inflammation-related Dexamethasone molecular signature in the liver of male rats, and higher susceptibility of male rats to anti-inflammatory actions of systemic Dexamethasone in a sepsis model [16]. This discrepancy probably reflects the significant difference in models and routs of GCs delivery. In addition, our recent analysis of sexual dimorphism in the molecular signature of topical glucocorticoid clobetasol propionate in human skin [32] did not reveal either the increased gene down-regulation or the increased number of inflammation-related DEGs in human male skin, suggesting that sexually dimorphic effects of GCs are tissue-specific.

Further, the analysis of skin atrophy induced by chronic topical FA showed higher sensitivity of female mice to atrophic effects of lower FA doses. This was rather unexpected in view of protective anti-atrophic effects of estrogen in naturally aging skin mostly studied in postmenopausal women [19, 33].

In our previous studies we discovered that REDD1, a stress-inducible inhibitor of mTOR/Akt, acts as atrophogene in the skin [12], and we were able to prevent steroid-induced skin hypoplasia in female mice by using pharmacological REDD1 inhibitors [23, 30]. We report here that there was a sexually dimorphic response to glucocorticoid FA in terms of REDD1 induction: in female skin, REDD1 expression was induced earlier and more efficiently, which correlated well with higher sensitivity of females to FA-induced atrophy. Interestingly, we also found that REDD1 knockout protected preferentially females but not males from skin hypoplasia. Together these results suggest that estrogen-enhanced glucocorticoid induction of REDD1 acts as a central driver of skin atrophy in females, but not in males, and that enhanced induction of REDD1 contributes to the greater sensitivity of females to steroid skin atrophy.

The effects of glucocorticoids and estrogens are mediated by their cognate receptors, GR and ER, which are well-known ligand-activated transcription factors from the family of steroid hormone receptors (SHR) [34]. Even though SHR–regulated transcription was initially studied as single receptor events, mediated by SHR homodimers [35], it is becoming evident that SHR crosstalk with each other and frequently co-regulate gene expression [34]. ER/GR crosstalk was extensively studied in hormone-dependent tissues such as uterus and breast, and in hormone-dependent cancers [34, 36–38]. Even though there has been a perception in the literature that GR and ER effects on gene expression are frequently reciprocal, careful literature analysis revealed that GR/ER interactions are much more complex and cell and gene context-dependent. Indeed, there is an extensive overlap in ER and GR chromatin binding; both hormones induce global reprogramming of chromatin landscape that affects DNA accessibly for both receptors [36, 38]. Moreover, upon co-activation, increased GR chromatin binding was observed at ER response elements, and at the same time, ER was associated with GR response elements, suggesting that ER and GR interact in a complex [34, 36, 38]. Further, analysis of the global effects of estrogens and GCs on transcriptome revealed similar effects of both hormones on individual gene expression [36, 39, 40]. There is also experimental evidence suggesting direct physical ER-GR interaction on protein-protein level [37], which further supports the results of global transcriptome analysis.

REDD1 is a known target gene for GR with multiple GRE in its promoter [21–23]. We report here that REDD1 is also a target gene for ER, and that its expression is cooperatively regulated by estrogens and glucocorticoids used in low, sub-threshold doses. Moreover, in correlation with the findings in other cell types, we observed that the steroid hormone effect on REDD1 expression depended on the functional activity of both ER and GR as the use of either of their antagonists blocked FA- and E_2_-dependent REDD1 induction.

In conclusion, we have demonstrated that there is sexual dimorphism in the adverse side effects of topical glucocorticoids but not in their anti-inflammatory effects. We found that female mice were more prone to glucocorticoid-induced skin atrophy than males due to the cooperative induction of REDD1 by estrogens and glucocorticoids. These observations are clinically important and suggest that our efforts focused on the development of safer GR-targeted therapy with topical glucocorticoids combined with anti-atrophogenes, including REDD1 inhibitors [23, 30] are more applicable for female patients suffering from inflammatory and hyperproliferative skin diseases and disorders.

## MATERIALS AND METHODS

### Chemicals, reagents, and antibodies

Fluocinolone acetonide (FA), E_2_, DHT, RU0486 (Mifepristone), and ICI182,780 (Fulvestrant) were from Sigma-Aldrich (St. Louis, MO, USA). Croton oil (CO) was from Santa Cruz Biotechnology (Dallas, TX, USA). We used antibodies against GR (Santa Cruz Biotechnology), ER (EMD Millipore, Danvers MA, USA), REDD1 (Proteintech Group, Rosemont, IL, USA), GAPDH (Sigma-Aldrich), tubulin, and normal IgG (Cell Signaling, San Jose, CA, USA).

### Animals

The REDD1 KO breeder mice in F1 C57BL/6 × 129S genetic background were kindly provided by Quark Pharmaceuticals Inc. (Newark, CA, USA); wild-type (WT) isogenic C57BL/6 × 129S breeder mice were obtained from Taconic (Germantown, NY, USA). Both colonies were bread at Northwestern University vivarium.

### Ear edema test

For evaluation of the anti-inflammatory effect of glucocorticoid FA, we used the ear edema test [19, 7]. Seven-week-old WT mice of both sexes were pretreated with FA (0.06–1 μg in 20 μl of acetone) or vehicle (20 μl of acetone) administered to the back of the ear lobe 1 h before application of nonspecific contact irritant croton oil (5% solution in 20 μl of acetone). Mice were euthanized, and ears were harvested 9 h after the croton oil application; at this time point we observed maximum ear edema in B6 × 129 animals [12]. Five-millimeter ear punch biopsies were weighted to measure ear swelling as a readout for inflammation.

### GCs-induced skin atrophy and acute topical dorsal skin treatments

Seven-week-old mice in the telogen stage of the hair cycle were shaved on the back and 3 days later treated with FA or vehicle as described below.

### Acute treatment

Animals were treated with FA applied topically (0.1–2 µg/mouse) in 200 µl acetone to the back skin, control animals were treated with acetone. The skin was harvested in 6–48 h, and epidermis, mechanically separated from dermis, was used for RNA isolation as described [12].

### Chronic treatment to induce skin atrophy

Skin atrophy was induced by chronic FA treatment (0.1–2 µg every 72 h for 2 weeks), as previously described [12]. Mice were sacrificed, and skin was harvested 24 hours after the last FA treatment.

In all experiments, we used 3–4 mice/group; experiments were repeated 2–3 times. All animal experiments were performed according to protocols approved by Northwestern University Animal Care and Use Committee.

### Histological analysis and morphometry

Skin was fixed with formalin, and paraffin-embedded sections were stained with hematoxylin and eosin (H&E). Quantification of the epidermal and dermal adipose width (as the readout for skin thinning) was performed as described [12, 30]. At least 10 fields of view/slide in each of three individual samples/experimental group (30 images/ group) were analyzed using Axioplan2 microscope software (Carl Zeiss).

### Cells and treatments

Immortalized human keratinocyte cell line HaCaT was a kind gift of Dr. K. J. Green (Northwestern University, Chicago, IL, USA). The cells were maintained in DMEM medium supplemented with 10% fetal bovine serum (FBS), 100 U/mL penicillin, 100 µg/mL streptomycin. Before treatments, cells were switched to hormone-free medium (Phenol red-free DMEM with 10% charcoal-stripped serum) for 24 h and then treated with glucocorticoid FA, estradiol (E_2_) or dihydrotestosterone (DHT) for 6, 12 or 24 h, as described in Figure legends. In some experiments, cells were pre-incubated for 6 h with RU0486 (Mifepristone, 10 µM) and ICI182,780 (Fulvestrant, 1 µM) used as GR and ER inhibitors, respectively. E_2_ and FA were dissolved in ethanol, DHT in methanol, and inhibitors in DMSO.

### Western blot analysis

Whole-cell protein extracts were prepared as described [12], resolved on 4–15% gradient SDS-PAGE, and transferred to nitrocellulose membranes (LI-COR Biosciences; Lincoln, NE, USA). Membranes were blocked and incubated with primary antibodies overnight at 4°C, followed by IRDye^®^ secondary antibodies (LI-COR Biosciences). LI-COR Odyssey Imager was used for band visualization. The antibodies were used at concentrations recommended by their manufacturers. GAPDH and tubulin were used as loading controls.

### RNA preparation and quantitative PCR

RNA from epidermis was isolated with the RiboPure kit (Ambion/Life Technologies; Grand Island, NY, USA) and treated with TURBO™ DNase (Ambion). Reverse transcription was performed on 1 µg RNA, using random hexamers and M-MLV reverse transcriptase (Invitrogen/Life Technologies) as described [23]. The REDD1-specific primers were designed with NCBI Primer-BLAST (REDD1 forward primer: 5′-GGG CCG GAG GAA GAC TCC TCA TA-3′; REDD1 reverse primer: 5′-CTG TAT GCC AGG CGC AGG AGT TC-3′. Q-PCR with SYBR Green detection was performed on the Applied Biosystems^®^ 7000 Real-Time PCR instrument (Life Technologies). Each sample was tested in triplicate. The results were normalized to the expression of the housekeeping Rpl27 gene.

### Chromatin immunoprecipitation

ChIP was performed to assess ER loading on REDD1 EREs using EZ-Magna ChIP A/G ChIP Kit (Millipore, Darmstadt, Germany) per the manufacturer’s recommendations. The ER binding sites in human REDD1 promoter were predicted based on HOCOMOCO models of TF binding motifs using ChIP-sequencing and high-throughput systematic evolution of ligands by exponential enrichment (i.e., HT-SELEX) data [31]. HaCaT cells were grown for 36 h in Phenol red-free DMEM with 10% charcoal-stripped serum, and then treated with E_2_ (1 µM) for 24 h. Fold enrichment was calculated as 2^−∆∆Ct^, where ∆Ct = Ct (IP) − Ct (Input × DF) and ∆∆Ct = ∆Ct (IP) − ∆Ct (NS). IP indicates immunoprecipitation, DF indicates the dilution factor, and NS indicates IP with nonspecific IgG. Data are mean +/–standard deviation (*n* = 3). The sequences of 7 putative estrogen response elements in the REDD1 promoter region and primers used for ChIP are detailed in Supplementary Tables 1 and 2.

### Statistical analysis

Means and standard deviations were calculated using Microsoft Excel software (Microsoft, Redmond, WA, USA). The treatment effects in each experiment were compared by *t*-test using the GraphPad statistical software (La Jolla, CA, USA) or Microsoft Excel software. For differences between groups, *P* < 0.05 was considered significant. Experiments were performed two-three times. In all figures, the results of one representative experiment are shown as mean value ± SD.

## SUPPLEMENTARY MATERIALS



## Abbreviations

AR: androgen receptor
CO: croton oil
DHT: dihydrotestosterone
E_2_: 17β-estradiol
ER: estrogen receptor
ERE: estrogen responsive element
FA: fluocinolone acetonide
GAPDH: glyceraldehyde 3-phosphate dehydrogenase
GCs: glucocorticoids
GR: glucocorticoid receptor
GRE: glucocorticoid-responsive element
KO: knockout
mTOR: mammalian target of rapamycin
mTORC1: mTOR complex 1
REDD1: regulated in development and DNA damage 1
SHR: steroid hormone receptors
