# Supplementary material for: Sexual dimorphism in atrophic effects of topical glucocorticoids is driven by differential regulation of atrophogene REDD1 in male and female skin
Source: Oncotarget. 2020 Jan 28;11(4):409–18. doi: 10.18632/oncotarget.27445 (PMC6996908; doi:10.18632/oncotarget.27445)
Supplement: Supplementary file 1 [file oncotarget-11-409-s001.pdf]

# Sexual dimorphism in atrophic effects of topical glucocorticoids is driven by differential regulation of atrophogene REDD1 in male and female skin

## SUPPLEMENTARY MATERIALS

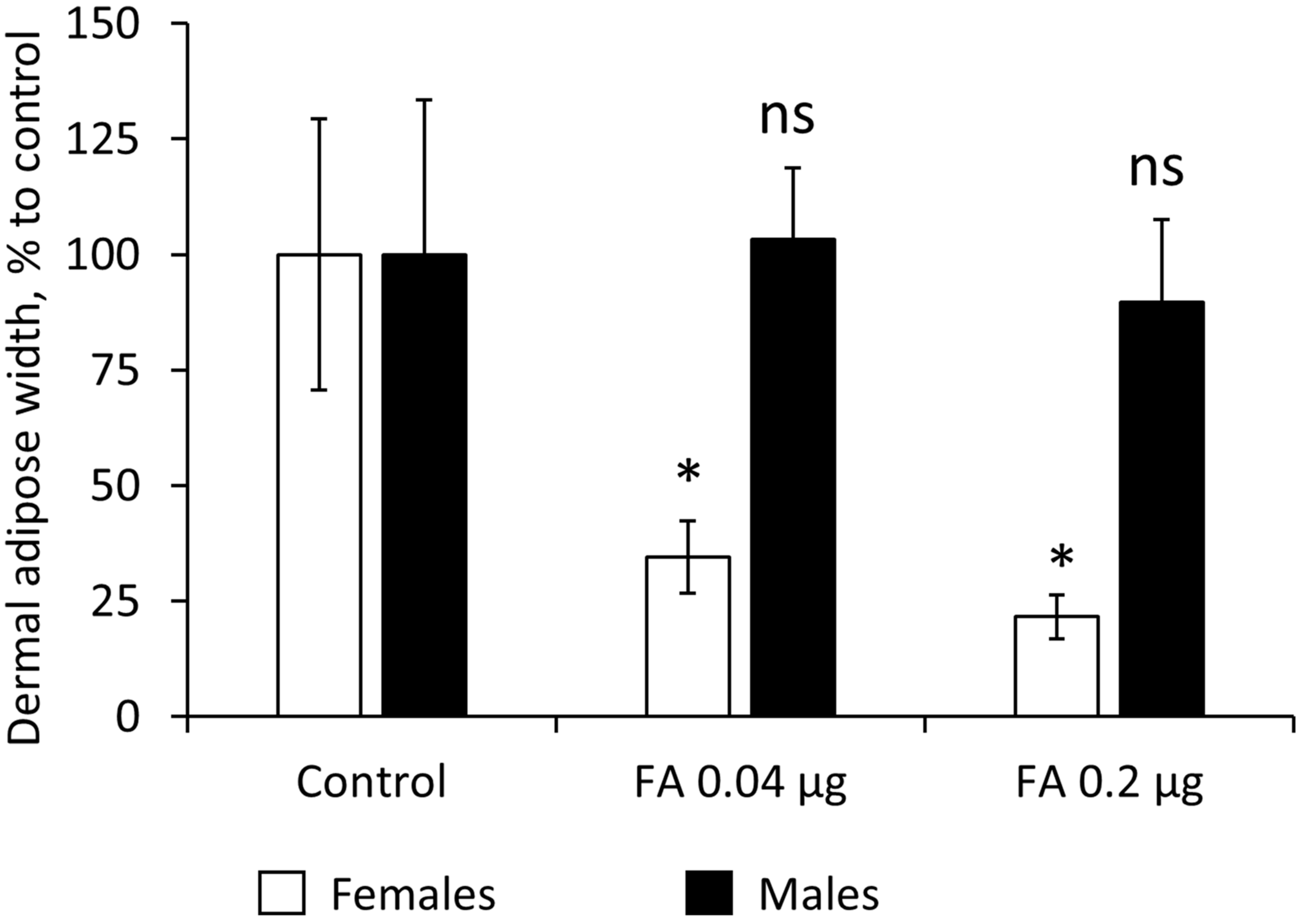


**Supplementary Figure 1: Higher sensitivity of dermal adipose to atrophic effects of glucocorticoids in females.** To induce dermal adipose atrophy, FA was applied topically to the dorsal skin of female and male B6x129 mice at lower doses for 2 weeks, as described in Materials and Methods. Acetone was used for a control treatment. The skin was harvested 24 h after the last treatment. H&E sections of the skin were used for morphometric analysis. Dermal adipose thickness is presented as % to the thickness of corresponding control adipose. Statistical analysis for the differences between control and FA-treated animals was done by unpaired two-tailed *t*-test. $^{*}p < 0.0001$; ns, not significant ($p > 0.2$).

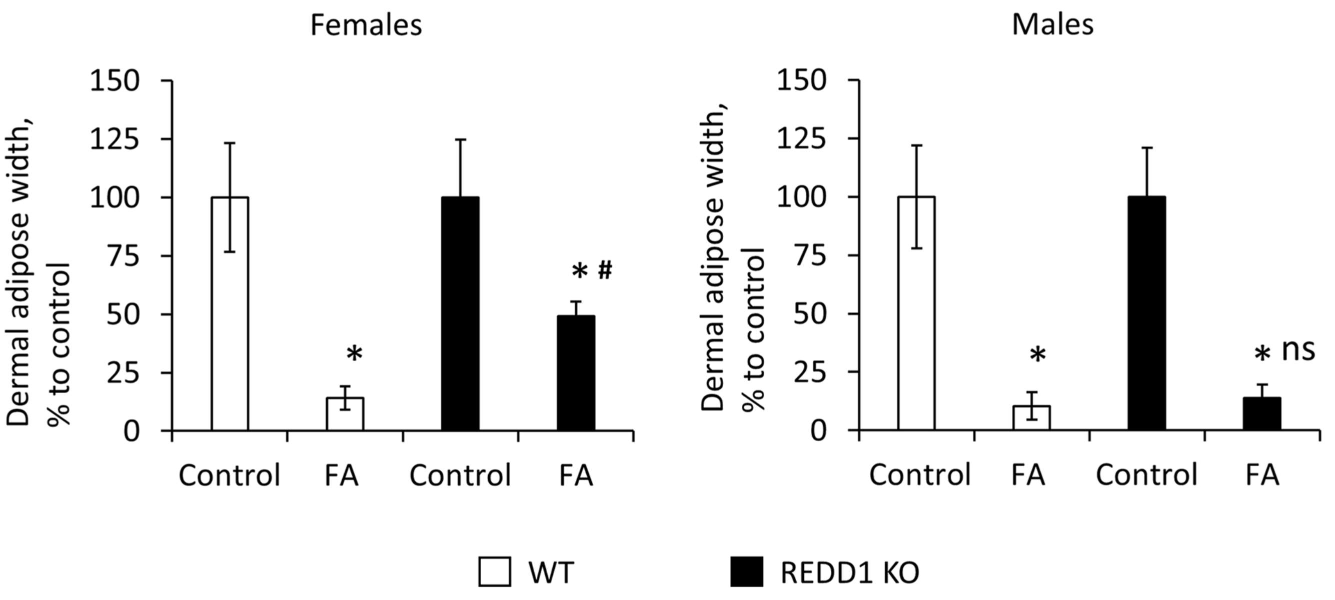


**Supplementary Figure 2: REDD1 KO female but not male mice are protected from GCs-induced dermal adipose atrophy.** To induce dermal adipose atrophy, glucocorticoid FA was applied topically (2 µg/animal) to the dorsal skin of female and male B6x129 and REDD1 KO mice for 2 weeks, as described in Materials and Methods. Acetone was used for a control treatment. The skin was harvested 24 h after the last treatment. H&E sections of the skin were used for morphometric analysis. Dermal adipose thickness is presented as % to the thickness of corresponding control adipose. The unpaired two-tailed *t*-test was used for statistical analysis: $^{*}P < 0.001$, for differences between the control and FA-treated animals; $^{\#}P < 0.001$, for differences between atrophy in WT and REDD1 KO females; ns ($P > 0.2$), for differences between atrophy in WT and REDD1 KO males.

## Supplementary Table 1: ERE sites in human REDD1 promoter

| Nomenclature | Sequence | Start position | End position | Region length, bp | Position, bp upstream from +1 |
|---|---|---|---|---|---|
| ERE1 | TGGGGGTTGCGGAGGGCAGTGGCCTG ACATCTCCTGCAAGTGTCATTTGTCACG CGAAAGCAAGCCTGACCCGAGGCAAGGCCC | 72272621 | 72272704 | 84 | 1500 |
| ERE2 | CCCTGGGCATCTGATGTAAGTTTGGCTC CCTCTTCCATCAGGGTCCTCCTGTCCCAG ACTGATAAACCTTTAGAGC | 72272740 | 72272815 | 76 | 1402 |
| ERE3 | GCAACCCTATAATAAACAAGTCTTTCC TTGATCCTC | 72273603 | 72273638 | 36 | 579 |
| ERE4 | AGCAGGAAGACACACCCATGCAGGT CACCTCTGT | 72272198 | 72272231 | 34 | 1986 |
| ERE5 | ACCTTGGCAGCTGCAGCCGCCGCGGATCCT TTCCAGAAAGGGGGCGTGGCGGTGGGTCG GGGTTCGACTGCGAG | 72273662 | 72273735 | 74 | 482 |
| ERE6 | TGGTTAGGTTCCTTGCCCGCCCCCGGGGCC CTGGGCATCTGATGTAAGTTTGGCTCCCTC TTCC | 72272712 | 72272775 | 64 | 1442 |
| ERE7 | CTCTTCCATCAGGGTCCTCCTGTCCCAGACTG ATAAACCTTTAGAGCAATAAAGGTCGAGTCC GTCTCAACCTCCACCCACCCTCAGCAACCAC | 72272769 | 72272862 | 94 | 1355 |

**Supplementary Table 2: Primers used for ChIP analysis of REDD1 promoter**

| Gene symbol | Primer sequence: sense/antisense (5′–3′) |
|---|---|
| *ERE1* | GGGGTGGGGATGGGGGTTGCG GATAGGCGGGCCTTGCCTCGGG |
| *ERE2* | ATCTGGTTAGGTTCCTTGCCCGCCC<br>GAGGGTGGGTGGAGGTTGAGACGG |
| *ERE3* | CCTAGCCTGGTCACGGGCTGTC<br>GCAGCTGCCAAGGTCCCCGAGG |
| *ERE4* | CCCGCCCAAATCAGCCTGGAG GCTAGTTGTTCTTGGAGGGCTGGAG |
| *ERE5* | TTCCTTGATCCTCCCCTGCCGC CCGCCTCCATTGAGCCCCAGAAAG |
| *ERE6* | CCGAGGCAAGGCCC GCCTATC CAGTCTGGGACAGGAGGACCCTG |
| *ERE7* | CGGGGCCCTGGGCATCTGATG CTGGCCTGGCAGCAGTGCTCAGAG |
| *Scramble S1* | GGGGTGGGGATGGGGGTTGCG<br>GATAGGCGGGCCTTGCCTCGGG |
